# The Role of AI in Serious Games and Gamification for Health: Scoping Review

**DOI:** 10.2196/48258

**Published:** 2024-01-15

**Authors:** Daniel Tolks, Johannes Jeremy Schmidt, Sebastian Kuhn

**Affiliations:** 1 Department of Digital Medicine, Medical Faculty OWL Bielefeld University Bielefeld Germany; 2 Centre for Applied Health Science Leuphana University Lueneburg Lueneburg Germany; 3 Institute for Digital Medicine University Clinic of Gießen und Marburg Philipps University Marburg Marburg Germany

**Keywords:** artificial intelligence, AI, games, serious games, gamification, health care, review

## Abstract

**Background:**

Artificial intelligence (AI) and game-based methods such as serious games or gamification are both emerging technologies and methodologies in health care. The merging of the two could provide greater advantages, particularly in the field of therapeutic interventions in medicine.

**Objective:**

This scoping review sought to generate an overview of the currently existing literature on the connection of AI and game-based approaches in health care. The primary objectives were to cluster studies by disease and health topic addressed, level of care, and AI or games technology.

**Methods:**

For this scoping review, the databases PubMed, Scopus, IEEE Xplore, Cochrane Library, and PubPsych were comprehensively searched on February 2, 2022. Two independent authors conducted the screening process using Rayyan software (Rayyan Systems Inc). Only original studies published in English since 1992 were eligible for inclusion. The studies had to involve aspects of therapy or education in medicine and the use of AI in combination with game-based approaches. Each publication was coded for basic characteristics, including the population, intervention, comparison, and outcomes (PICO) criteria; the level of evidence; the disease and health issue; the level of care; the game variant; the AI technology; and the function type. Inductive coding was used to identify the patterns, themes, and categories in the data. Individual codings were analyzed and summarized narratively.

**Results:**

A total of 16 papers met all inclusion criteria. Most of the studies (10/16, 63%) were conducted in disease rehabilitation, tackling motion impairment (eg, after stroke or trauma). Another cluster of studies (3/16, 19%) was found in the detection and rehabilitation of cognitive impairment. Machine learning was the main AI technology applied and serious games the main game-based approach used. However, direct interaction between the technologies occurred only in 3 (19%) of the 16 studies. The included studies all show very limited quality evidence. From the patients’ and healthy individuals’ perspective, generally high usability, motivation, and satisfaction were found.

**Conclusions:**

The review shows limited quality of evidence for the combination of AI and games in health care. Most of the included studies were nonrandomized pilot studies with few participants (14/16, 88%). This leads to a high risk for a range of biases and limits overall conclusions. However, the first results present a broad scope of possible applications, especially in motion and cognitive impairment, as well as positive perceptions by patients. In future, the development of adaptive game designs with direct interaction between AI and games seems promising and should be a topic for future reviews.

## Introduction

### Background

Artificial intelligence (AI) and serious games are both relevant topics in the health sector, and the body of studies and literature is continuously growing. Interestingly, in terms of the research landscape, the 2 topics are not connected; rather, existing research views them independently.

The use of games for educational and serious purposes is nearly as old as the history of humankind and is an integral part of our culture [1]. In 1970, Abt [2] used the term “serious games” for the first time in his book with the same name. Sawyer and Smith [3] take a broad definition and consider serious games as “any computerized game whose chief mission is not entertainment and all entertainment games which can be reapplied to a different mission other than entertainment.” What serious games have in common is that they pursue a concrete (pedagogical) intention and aim to provide information on a specific topic (eg, health) that is accessible in an entertaining and interactive way to deepen competencies or to achieve a change in behavior [4].

Serious games for health can be used in the fields of medical diagnostics, therapy, and prevention, as well as health promotion and medical or patient education [5]. From a didactic and learning psychology perspective, the effect of serious games is based on the integration of the motivating and multimedia aspects of computer and video games. Serious games can increase engagement, motivation, enthusiasm, and interest [6,7]. There are several existing use cases in health contexts [8-11]. One example is the game EndeavorRx. In 2020, the US Food and Drug Administration permitted its marketing as the first game-based digital therapeutic device to improve attention function in children with attention-deficit/hyperactivity disorder (ADHD) [12]. The game Re-Mission was developed for children with cancer and showed good results regarding compliance and the understanding of disease-related issues in the target group [13]. EMERGE is a simulation game that recreates an emergency department in real time to improve the clinical reasoning skills of physicians [14].

Next to serious games, gamification has emerged as a major trend in the health sector, which is reflected in a growing number of publications, including several meta-analyses [15-17]. The most used definition of this concept is “the use of game design elements in non-game contexts” [18]. The motivational effect of the game elements can be explained in different ways. Sailer et al [19] established the link between various gamification elements (eg, points, leaderboards, and badges) and the self-determination theory proposed by Ryan and Deci [20]. As a theory of motivation, this defines three universal psychological basic needs that determine human action: (1) competence, (2) autonomy, and (3) social inclusion. If ≥1 of these needs are addressed (eg, through gamification elements), this has positive effects on behavior and its determinants [19]. In the health sector, there are numerous studies that have demonstrated the effects of using gamification on motivation, performance, engagement, health, and well-being status [5,21,22].

According to Westera et al [23], computer games have been linked with AI since the first computer was programmed to play chess [24]. New AI methods have been used in computer games, for instance, to generate levels, scenarios, and storylines; to balance complexity; or to add intelligent behaviors to nonplayer characters (NPCs) [25]. However, over the years, various authors have pointed at the marginal penetration of academic game AI methods in industrial game production [26]. AI techniques will become indispensable to coordinate the ever-growing complexity and dynamics of games [23]. AI-driven adaptation and assessment systems are used to offer learner-centered environments [27]. As an example, NPCs controlled by AI can adapt to the behavior of the gamer and can enrich immersive and challenging experiences within the game play.

When transferring these principles to health care, the interaction between AI and games could provide a benefit, especially in the management of chronic diseases, which most game designs already target. The possibility to quickly adapt to new game-generated data or performance and provide live feedback could lead to more individual and thus more patient-centered game design in both illness detection and treatment. This could increase motivation and engagement for patients, leading to higher therapy adherence through more personal involvement. The vast body of evidence in the field of serious games and gamification, along with the growing body of evidence in the use of AI, may thus form a new field of research.

### Scope

Therefore, this scoping review sought to generate an overview of the currently existing literature on the interaction of AI and game-based approaches in health care. At this point, to the best of our knowledge, this is the only review that targets this interaction.

The primary objective was to analyze the current body of evidence based on (1) the disease or health issue being evaluated, (2) the process of care in which these projects are located, and (3) the kind of AI and type of game-based approach used and the interaction of both techniques.

A secondary objective was to obtain an overview of publications on the interaction of AI and game-based approaches such as serious games, gamification, commercial games, and game periphery. Another secondary objective was to analyze the quality of the existing studies in this field regarding their grade of evidence and the conducted study types.

## Methods

### Overview

**Figure 1:**
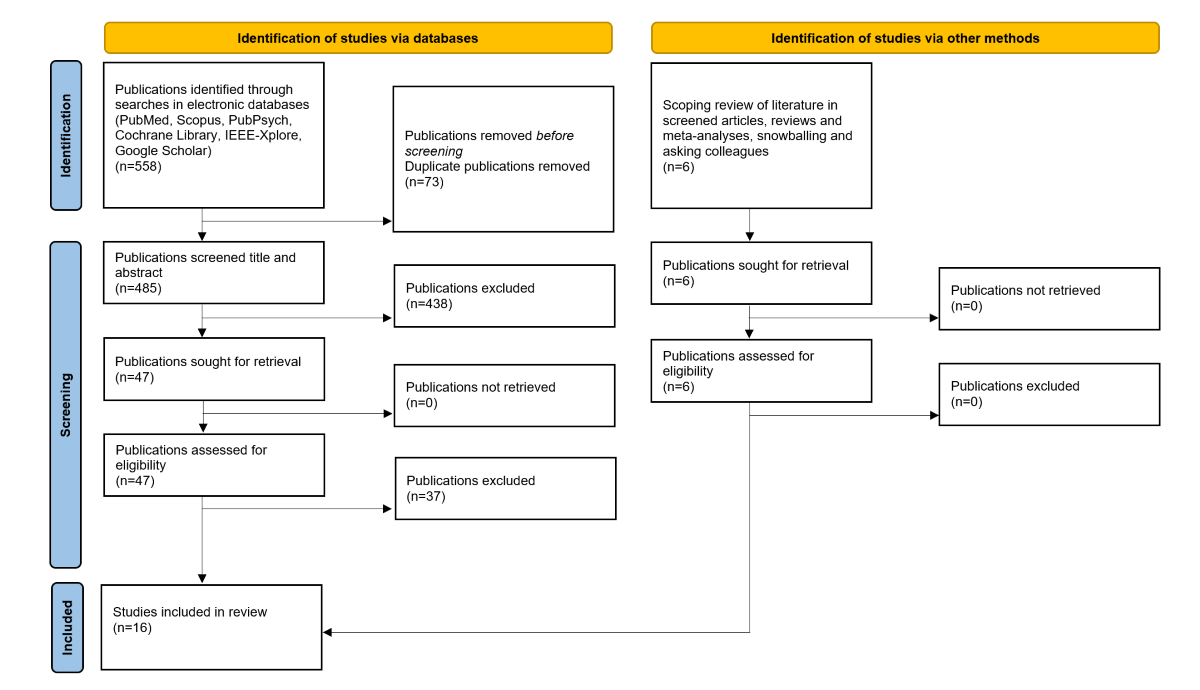
PRISMA-P (Preferred Reporting Items for Systematic Review and Meta-Analysis Protocols) flow diagram.

For this scoping review, we applied the PRISMA-P (Preferred Reporting Items for Systematic Review and Meta-Analysis Protocols; Figure 1) guidelines [28]. Furthermore, we used the recommendations of the Cochrane Consortium for conducting systematic reviews and the RefHunter website as guidance [29].

Before starting the review process, we defined the inclusion and exclusion criteria (Textbox 1).

**Inclusion and exclusion criteria.**

**Inclusion criteria**

- Article type: original study, journal article, or conference paper
- Article scope: articles report the use of artificial intelligence (AI), machine learning, and deep learning in combination with game-based approaches (serious games, gamification, and game-based-learning)
- Health profession: medicine
- Area of application: articles that conducted research in the field of education, therapy, and health
- Language: English
- Publication period: last 20 years

**Exclusion criteria**

- Article type: opinion, commentary, or letter to the editor
- Article scope: not related to AI and game-based approaches
- Health profession: other than medicine
- Area of application: not related to health and medicine
- Language: not in English
- Publication period: published >20 years ago

For this review, we conducted the following steps:

1. Literature search
2. Title and abstract screening
3. Content screening
4. Further in-depth screening (snowballing method and asking colleagues)

### Step 1: Literature Search

We applied the search terms primarily in the database PubMed on February 2, 2022. We tested and honed different search terms and Boolean operators (Multimedia Appendix 1) until sufficiently fitting results seemed to have been obtained (n=305). The final search term was defined as follows:

[“game” OR “gamification”] AND “artificial intelligence”

The search was extended to more open databases to assess studies that target AI and serious games in medicine-related research areas or interprofessional approaches that may include medical professions and in more technically oriented databases such as IEEE Xplore [30] to include papers from informatics and engineering with a focus on technical issues.

The same search term ([“game” OR “gamification”] AND “artificial intelligence”) AND “artificial intelligence”) was used for IEEE Xplore (n=98), Cochrane Library [31] (n=25), and PubPsych [32] (n=89). In Scopus, the search term used in the other databases showed fewer results and were modified to extend the range of hits (“serious” AND “game” AND “artificial intelligence”; n=41).

In addition, we conducted a search in Google Scholar [33]. However, the results from Google Scholar were not precise enough for inclusion in a review, which is consistent with the results of several studies [34-36].

After deduplication using Rayyan software (Rayyan Systems Inc), the combined search in these databases identified 545 (97.7%) publications out of the initial 558 identified. We then performed a manual deduplication, which resulted in 60 (11%) of the 545 publications being excluded; thus 485 (89%) publications remained (Multimedia Appendices 1 and 2; Multimedia Appendix 3 [37-51]).

### Step 2: Title and Abstract Screening

For the second step of the scoping review (title and abstract screening), we used Rayyan software. The results from the literature search were transferred to the citation software Zotero (version 5.0.85; Rayyan) and to Rayyan software [52]. This software automatically identified duplicates. After iterative deduplication, the publications were subjected to manual screening. The first screening step was conducted using Rayyan and permitted publication inclusion based on their titles and abstracts. Given the volume of the publications to be screened, the title and abstract screening was distributed among 2 authors of this paper (JS and DT). To ensure the uniformity of the screening, the authors conducted several training sessions in Rayyan with the coreviewers. In addition, the authors randomly double-checked some of the excluded publications (25/485, 5.2%) to warrant the consistency of the screening by the other reviewer. This step was conducted independently by both researchers and was followed by a discussion of the results between the 2. As all analysis steps were conducted independently by the 2 researchers, a discussion of differently categorized literature (marked as “Conflict” in Rayyan) and subsequent adaptation were necessary in this step. Overall, only a few adjustments were necessary, and a good agreement between the 2 researchers could be reached. Possible conflicts and all included articles were discussed with a third team member (SK). The data generated by Rayyan can be found in Multimedia Appendix 2.

### Step 3: Content Screening

After the primary screening, full-text publications were screened by the 2 lead authors. Toward this end, a table was prepared to compile relevant information (Textbox 2).

**Information compiled for full-text screening.**

**Relevant details obtained**

- Authors, year, title, journal, and digital object identifier (DOI)
- Study type (according to Röhrig et al [53])
- Population, intervention, comparison, and outcomes (PICO) criteria
- Subject (topic of the study)
- Level of evidence (according to the Oxford Centre for Evidence-Based Medicine: Levels of Evidence [54])
- Disease or health issue
- Level of care (prevention, diagnostics, therapy, rehabilitation, nursing, organization or monitoring, and other [55])
- Game variants (serious games, gamification, games, and game controller or periphery)
- Artificial intelligence (AI) technology (machine learning, deep learning, and AI [not further specified])
- Function type (promoting health literacy, analysis and cognition, indirect intervention, direct intervention, documentation of health and medical history, organization and administration, and purchasing and supply) [56]

Some of the studies (4/16, 25%) showed an overlap among different categories (eg, in level of care). In these cases, double classifications were performed. All eligible studies were categorized and coded in detail (Multimedia Appendix 3 [37-51]).

### Step 4: Further In-Depth Screening (Snowballing Method and Asking Colleagues)

After conducting the scoping review, we additionally used the “snowballing” approach described by Greenhalgh and Peacock [57], who have stated that in reviews of complex and heterogeneous evidence, formal protocol-driven search strategies may fail to identify important evidence. Informal approaches such as browsing and asking colleagues can substantially increase the efficiency of search efforts. Snowballing methods such as pursuing references of references and electronic citation tracking are very useful for identifying high-quality sources in obscure locations. Therefore, to validate the results of the review, the 2 reviewers searched the literature references used in meta-analyses, reviews, and papers that were closely related to the topic of the search. In addition to using the snowballing method, the method of asking colleagues, as recommended by Greenhalgh and Peacock [57], was applied as a last step.

## Results

### Overview

When applying the aforementioned search terms, we initially identified 335 studies on the topic of games and the use of AI in health care in the last 20 years. The subsequently performed step of title and abstract screening reduced the number of the initially identified studies from 335 to 47 (14%). In the next step, assessing the actual full-text literature, of the 47 papers, 3 (6%) were excluded because their full text was not in English, and the aforementioned inclusion and exclusion criteria were applied to the remaining 44 (94%). After the full-text screening, 10 (23%) of the 44 papers met all inclusion criteria. Using the snowballing method, 1 additional paper could be identified. Asking colleagues revealed 5 additional papers, which led to an overall total of 16 eligible papers (Figure 1). Not all criteria showed hits (eg, function type showed hits only in 2 categories, whereas level of care showed no hits in nursing).

### Categories

#### Overview

The eligible papers showed a clear emphasis on certain categories (Table 1). Regarding the targeted diseases, the field of motion impairment was investigated the most (5/16, 31%). Cognitive impairment was targeted in 19% (3/16) of the studies, phantom limb pain or limb absence in 19% (3/16), rheumatoid arthritis in 13% (2/16), cancer in 6% (1/16), and ADHD in 6% (1/16). The primary focus on rehabilitation (10/16, 63%) was the most compelling. Of the 16 studies, 5 (31%) took place in the field of prevention, 4 (25%) in the field of diagnostics, and 1 (6%) in a nonrehabilitation therapeutic context (4 double assignments).

Most of the studies (12/16, 75%) applied machine learning as the AI technology, and 13% (2/16) used deep learning, whereas the remaining studies (2/16, 13%) did not specify the AI technology. Most of the studies (11/16, 69%) used a serious game, whereas 19% (3/16) used a commercial games approach. Despite the highly increased use of gamification in health and education, of the 16 studies, only 1 (6%) specifically used gamification to improve the motivation of patients, and 1 (6%) used game design–like interactions.

We further clustered and outlined the eligible papers according to the targeted disease (Table 2). A more detailed description of every included publication with a more specific outline of the use of AI and game variant can be found in Multimedia Appendix 4 [37-51].

**Table 1:** Categories.

Category		Studies, n (%)
**Disease or health topic (n=16)**		
	Motion impairment	5 (31)
	Phantom limb pain or limb absence	3 (19)
	Cognitive impairment	3 (19)
	Rheumatoid arthritis	2 (13)
	Cancer	1 (6)
	Attention-deficit/hyperactivity disorder	1 (6)
	Other	1 (6)
**Function type (n=16)**		
	Direct intervention	9 (56)
	Analysis and cognition	7 (44)
**Level of care (n=20^a^)**		
	Prevention	5 (25)
	Diagnostics	4 (20)
	Therapy	1 (5)
	Rehabilitation	10 (50)
**AI^b^ technology (n=16)**		
	Machine learning	12 (75)
	Deep learning	2 (13)
	AI (not further specified)	2 (13)
**Game variant (n=16)**		
	Serious games	11 (69)
	Gamification	1 (6)
	Games	3 (19)
	Game periphery	1 (6)

^a^A total of 4 studies showed an overlap between prevention and diagnostics and were double classified, resulting in an overall total of 20 studies.

^b^AI: artificial intelligence.

**Table 2:** Overview of included papers, structured by disease or health topic.

Authors; year			Target group (participants, n)		Subject		Study design		Level of evidence^a^		Level of care		Function type		AI^b^ technology		Game variant
**Disease or health topic: motion impairment**																	
	Yeh et al [43]; 2014	Patients (48)		Noninvasive balance training		Case control study		3b		Therapy		Direct intervention		Machine learning		Games	
	Lyu et al [44]; 2019	Healthy individuals (8)		Electromyography-controlled knee exoskeleton		Quantitative, proof of concept		5		Rehabilitation		Analysis and cognition		Deep learning		Games	
	Nasri et al [45]; 2020	Patients (15)		Real-time hand gesture recognition		Case series		4		Rehabilitation		Direct intervention		Deep learning		Serious games	
	Burdea et al [42]; 2021	Healthy individuals (2)		Game controller–based telerehabilitation		Proof of concept		5		Rehabilitation		Direct intervention		AI		Game controller or periphery	
	Zhang et al [46]; 2021	Patients (5)		Gait analysis and waist motion capture		Case series		4		Rehabilitation		Direct intervention		Machine learning		Serious games	
**Disease or health topic: phantom limb pain or limb absence**																	
	Ortiz-Catalan et al [47]; 2016	Patients (14)		Phantom motor execution		Quantitative clinical trial		4		Rehabilitation		Direct intervention		Machine learning		Games	
	Lendaro et al [48]; 2019	Patients (4)		Phantom motor execution		Quantitative clinical trial		4		Rehabilitation		Analysis and cognition		Machine learning		Serious games	
	Kristofferson et al [49]; 2021	Patients (4)		Prosthesis system		Explorative study		4		Rehabilitation		Direct intervention		Machine learning		Serious games	
**Disease or health topic: cognitive impairment**																	
	Valladares-Rodriguez et al [50]; 2018	Healthy individuals or patients (16)		Early detection of mild cognitive impairment		Proof of concept		5		Prevention or diagnostics		Analysis and cognition		Machine learning		Serious games	
	Valladares-Rodriguez et al [51]; 2019	Patients (74)		Early detection of mild cognitive impairment		Case series		4		Prevention or diagnostics		Analysis and cognition		Machine learning		Serious games	
	Jung et al [37]; 2019	Patients (12)		Mini-Mental State Examination		Case series		4		Rehabilitation		Direct intervention		Machine learning		Serious games	
**Disease or health topic: cancer**																	
	Good et al [39]; 2014	Registered players (1077)		Gene Selection for breast cancer survival prediction		Quantitative study		5		Prevention		Analysis and cognition		Machine learning		Serious games	
**Disease or health topic: attention-deficit/hyperactivity disorder**																	
	Keshav et al [40]; 2019	Patients (7)		Digital attention-related augmented reality game		Case series		4		Prevention or diagnostics		Analysis and cognition		AI		Serious games	
**Disease or health topic: rheumatoid arthritis**																	
	Varga et al [38]; 2021	Healthy individuals (7)		Virtual arthritis rehabilitation app		Proof of concept		5		Rehabilitation		Direct intervention		Machine learning		Serious games	
	Varga et al [58]; 2022	Patients (10)		Virtual arthritis rehabilitation app		Case series		4		Rehabilitation		Direct intervention		Machine learning		Serious games	
**Disease or health topic: other**																	
	Pinto et al [41]; 2019	Older adults (11)		Active and assisted living for monitoring daily life activities		Case series		4		Prevention or diagnostics		Analysis and cognition		Machine learning		Gamification	

^a^According to the Oxford Centre for Evidence-Based Medicine: Levels of Evidence [54].

^b^AI: artificial intelligence.

#### Motion Impairment

Almost one-third of the studies (5/16, 31%) targeted the objective of motion impairment. Studies included upper- and lower-limb rehabilitation with a broad range of possible medical indications, ranging from poststroke to vestibular dysfunction. Games were used to enhance motivation and provide a user-friendly at-home training experience. Some of the studies (5/16, 31%) achieved this through an integration of virtual reality and artificial reality. AI was integrated in different ways. Some of the studies (4/16, 25%) used games as a training tool and then analyzed and classified the collected data with AI. Other studies (3/16, 19%) first processed sensor data via AI to improve the quality of an associated game. Direct interaction between the AI and the games component was shown in 2 (40%) of the 5 studies, in which AI adapted the game design and difficulty to the ability level of the patient.

Only 1 (20%) of the 5 studies tested the design using a control group analyzing patient improvements in clinical parameters. All other studies demonstrated the functionality and usability of their technical approach in pilot studies.

#### Phantom Limb Pain or Limb Absence

Of the 16 studies, 3 (19%) targeted the topic of phantom limb pain or limb absence, where a game environment can support at-home therapy and provide enhanced visual feedback. Of the 3 studies, 2 (67%) by the same research group targeted phantom motor execution with similar approaches. Machine learning was used to improve the quality of electromyography sensor data and thus provide better data input for training. Different training methods in the spectrum of virtual reality and augmented reality and serious games were tested. Of the 3 studies, 1 (33%) focused on ethnographic user–type analysis, and 1 (33%) effected a decrease in phantom pain. The third study tested a machine learning–aided prothesis, comparing 2 different training approaches—1 conventional and 1 via a serious game—to collect electromyography data. Testing was only conducted on 4 patients; however, the results were insignificant.

#### Cognitive Impairment

In cognitive impairment, the included studies used a set of games covering different cognitive functions as diagnostic instruments. Data were then processed by machine learning techniques to further improve outcome quality. Of the 3 studies, 1 (33%) focused on evaluating patients with cognitive impairment after stroke. Scores acquired from a game set were analyzed by AI and compared with the clinically widely used Mini-Mental State Examination (MMSE) [37]. Of the 3 studies, 2 (67%) used a game set for predicting the future development of mild cognitive impairment, using AI to automatically distinguish between healthy individuals and individuals who were possibly affected. In both fields, pilot studies were conducted with patients, showing high motivation to participate and good usability of the game sets.

#### Rheumatoid Arthritis

In rheumatoid arthritis, a serious game for hand rehabilitation was developed. Neural networks for processing data and machine learning for testing the accuracy of hand movements for individually adapting difficulty were integrated. Two small pilot studies, 1 with healthy individuals and 1 with patients, showed high accuracy of the machine learning algorithm and good usability, whereas clinical benefits have not yet been measured [38].

#### Cancer

In cancer, a crowdsourcing campaign was set up via an open web-based game that captured inputs from players regarding their estimation of 5 specific genes, which can be used as predictors of breast cancer survival. Gene selections were processed by machine learning to identify the best prediction models. When only including inputs from people with a self-proclaimed Doctor of Medicine degree, a Doctor of Philosophy degree, or expertise in cancer, the resulting models performed similarly to clinically established gene sets [39].

#### ADHD Severity

A set of smartglasses was developed to assess ADHD severity through playing an attention-related augmented reality game designed as a social-emotional communication aid. AI was used to analyze video and audio as well as affective and behavioral data and provided users with in-game rewards based on their performance. The study showed significant correlation of the game score to validated clinical gold standard assessments for ADHD [40].

#### Other

To improve the prevention of cognitive and physical decline, an at-home innovative system consisting of remote monitoring and neurocognitive games was developed. Feedback to the user, including badges or benefits for real-life events, is provided via machine learning analysis. Older adult users indicated “great acceptability” of the system [41].

## Discussion

### Principal Findings

Currently, there are only a limited number of studies involving a combination of game-based methods and AI in health. Almost one-third of the included studies (5/16, 31%) were centered on addressing motion impairment. The primary emphasis of the research was on rehabilitation. In addition, most of the studies (9/16, 56%) focused on prevention and diagnostics. In terms of AI technology, machine learning was the most commonly used approach (12/16, 75%). Furthermore, serious games were used in most of the studies (11/16, 69%).

When analyzing the studies by disease category, most of the studies (5/16, 31%) used a rehabilitation approach for different aspects of motion impairment (eg, in poststroke conditions, phantom limb pain or limb absence, and rheumatoid arthritis). In this field, studies have a focus on providing individual, at-home, and complex training opportunities for improving motoric limb function, in which therapeutic concepts rely on long-term and self-guided exercising. Games take the role of a training tool, enhancing at-home training motivation and providing multidimensional and exercises compared with the current standard of care. In addition, in some of the studies (5/16, 31%), the integration of virtual reality and augmented reality provided an immersive experience. The role of AI in this context is diverse, sometimes to analyze and classify collected data to improve game setup and level, sometimes to analyze data resulting from game play itself.

In a second cluster, studies for neurological diseases, including those handling cognitive impairment in older adults as well as 1 study for ADHD in younger patients, there was a clear focus on diagnostic evaluations. Here, different sets of games were used to assess various cognitive subdomains, with AI processing these different data inputs and calculating scores and predictions. The advantages in this field are the wide range of possible game designs and the feasibility to play these games individually at home. This could reduce health professionals’ time in assessing cognitive function during face-to-face visits or supplement them by enabling longitudinally acquired data sets and trajectories. The first results show promising results in comparison with standard clinical scores obtained using, for instance, the MMSE.

The direct interaction between the games approach and AI technology was only described in 3 (19%) of the 16 studies. Most of the time, the 2 entities follow each other, with the AI technology not analyzing live in-game playing data. However, direct interaction holds a promise of benefit through an AI-enabled assessment of the patient’s ability during game play and individualized live adjustments of game design and difficulty. Examples using this approach showed good technical functioning and positive user feedback [42]. Even so, the limited number of published studies suggest that the potential of this integrated approach has so far not been fully used yet. This is rather surprising, given the fact that the direct link between AI and games is widely prevalent in the commercial games sector. The reasons for this are purely speculative. The transition of findings from 1 field to another is still pending, perhaps because studies in the commercial games field have a different scope than those in medicine and health. Another reason could be the resource-intensive nature of research. However, the future potential of this interaction seems promising, with the stimulation of user motivation by game design and gamification elements and with AI being used to process large and multimodal data sources and to perform individualized adaptations.

When analyzing further categories, our review shows that the studies so far have produced very limited quality evidence (all studies have an evidence level of 4 or 5, except for 1 study that has an evidence level of 3b), with most of the studies presenting either a rather technical proof of concept (15/16, 94%) or performing usability testing with a small sample size of healthy individuals and patients (14/16, 88%). Higher-quality studies with control groups and end points focusing on specific clinical outcomes are missing.

Of note, the research field is still young. All studies were conducted in the last 8 years, with 13 (81%) of the 16 studies being published in the last 3 years. All research settings however bear the potential of conducting higher-quality studies with bigger sample sizes and specific medical outcomes in the near future.

However, the studies in this review already show promising results, with overall well-functioning technical implementation of the game elements and high accuracy and usefulness of the AI integration. From the patients’ and healthy individuals’ perspective, generally high usability, motivation, and satisfaction were found, mostly assessed by established usability questionnaires and qualitative interviews. This is an encouraging perspective for the future because individualized patient-driven at-home diagnostic and therapeutic approaches are increasingly relevant in all fields of medicine.

All 16 studies identified in this review have a relatively low level of evidence (3b: n=1, 6%; 4: n=10, 63%; and 5: n=5, 31%). The risks of bias in these studies are multifaceted. Pilot studies, often conducted to assess the feasibility of a full-scale study, typically featured small sample sizes and often lacked rigorous methodology, randomization, and blinding procedures. As a result, they are susceptible to a range of biases, including selection bias, performance bias, and detection bias. Studies were characterized by weaker methodologies, which can lead to biases in data collection, analysis, and reporting. Nonrandomized studies were prone to selection bias, confounding, and other methodological flaws. The high heterogeneity of the identified studies encompassed a wide range of disease or health issues, populations, and interventions. This heterogeneity makes it challenging or impossible to integrate data and limits overall conclusions.

### Limitations

First, as described earlier, the field of research is still very interdisciplinary, and the studies carried out are very diverse based on the vast variety of game-based approaches and therapeutic interventions.

This review only covered original studies in English, which were found in the PubMed, Scopus, IEEE Xplore, Cochrane Library, and PubPsych databases and published in the last 20 years. Although these are widely recognized and commonly used databases in the field of health care research, restricting the review to these 5 databases may have resulted in the exclusion of relevant studies published in other databases owing to this high interdisciplinarity. However, efforts have been made to minimize this limitation using comprehensive search strings, snowballing, and asking colleagues to identify additional relevant literature. In addition, this review also includes interdisciplinary databases such as the more technical-oriented IEEE Xplore and the more pedagogical-oriented PubPsych.

It especially remains unclear whether all projects conducted especially with a more technical focus have been published in scientific journals at all. For future reviews, a more holistic approach should be taken to assess more results from projects that may not have been included in a publication.

In addition, there might be a lack of awareness that research in the engineering, gaming, and fitness spectrum has a direct connection with health-related issues. Thus, it seems possible that certain publications were not fully covered by our already broad search strategy or that promising interventions have not been related to health care yet. This should be mitigated in future studies, considering the growing attention to this young research field.

Another limitation is that this review focused on therapeutic medical interventions rather than on health interventions. AI and game-based approaches in the field of prevention and health promotion have not been included, although this is an important aspect of population health. Game-based approaches especially are used a lot in this field to reach the target groups [8,9,21,22,59-61].

### Future Directions

In the near future, the potential of games, which is already established in the commercial games sector, should be applied to the field of serious games and AI. Adaptive game design can be suitable in health care to improve the intervention outcome via AI-driven health care games that assess the skills level of the patient and adapt the difficulty in feedback loops, which could lead to a better harmonization with traditional therapy sessions. NPCs could be used as virtual patients or other health care personnel or relatives to simulate the interprofessional working environment and to improve the interaction and communication with virtual patients [26,27,34].

Finally, the integration of AI and games should carefully consider the ongoing discussions regarding ethical, moral, and data protection issues. In particular, studies describing ethical issues using game-based approaches are scarce [62,63].

Analyzing the currently limited evidence with promising future possibilities in study design and quality, as well as a dynamic research field, it seems, at this stage, that another review should be conducted in the next few years to assess this rapidly growing research field.

## Appendix

Multimedia Appendix 1 Search term protocol.

Multimedia Appendix 2 Rayyan data of the review process of all studies.

Multimedia Appendix 3 Categorization of all eligible studies.

Multimedia Appendix 4 Detailed descriptions of all included studies.

Multimedia Appendix 5 PRISMA-P-Checklist.

## Abbreviations

ADHD: attention-deficit/hyperactivity disorder
AI: artificial intelligence
MMSE: Mini-Mental State Examination
NPC: nonplayer character
PRISMA-P: Preferred Reporting Items for Systematic Review and Meta-Analysis Protocols
