# Supplementary material for: The Role of AI in Serious Games and Gamification for Health: Scoping Review
Source: JMIR Serious Games. 2024 Jan 15;12:e48258. doi: 10.2196/48258 (PMC10825760; doi:10.2196/48258)
Supplement: Multimedia Appendix 1 [file games_v12i1e48258_app1.docx]

# Search Terms Protocol

**Including**

- Artificial intelligence
- Machine learning
- Deep learning
- Game, game-based learning, serious games, gamification
- Health, medicine
- (Medicine, Public Health) GoogleScholar und Co.
- All study types
- Education
- Therapy
- Projects
- Journals, conference papers
- Last 20 years
- English language

**Excluding**

- Opinions
- Commentary
- Letter
- Not related to health

**Cluster**

- Therapy
  - phantom limbs pain 2
  - cognitive impairments (detection/assessment: 1) 3
  - autism
- Medium
  - Virtual reality
  - Augmented reality
  - Mobile game
- Topics
  - Public health
  - Decision making
  - Machine learning 2
- Study type
  - Clinical trial
  - User interaction study
  - Preliminary study
  - Phase 3 diagnostic evaluation
  - Initial Proof of Concept
  - Review 2
  - Case

**Search terms - Boolean Operators**

- "serious games" AND "artificial intelligence" "medical education"

2

- "serious games" AND "artificial intelligence" "medical education"

12

- "games" AND "artificial intelligence"

221

- "game" AND "artificial intelligence"

313

- ["game" OR "gamification"] AND "artificial intelligence"

326

- ["game" OR "gamification"] AND "artificial intelligence" AND "medicine"

42

- ["game" OR "gamification"] AND "artificial intelligence" AND "machine learning"

52

- ["game" OR "gamification"] AND "artificial intelligence" AND "health"

52

- "game-based learning" AND "artificial intelligence"

3

- "game-based" AND "artificial intelligence"

11

**Other search engines**

https://ieeexplore.ieee.org/Xplore/home.jsp

- "game" OR "gamification"] AND "artificial intelligence"
  98

https://www.cochranelibrary.com/

- "game" OR "gamification" AND "artificial intelligence"
  25

https://www.scopus.com/

- "serious"  AND  "game"  AND  "artificial intelligence" 
  558

https://www.pubpsych.eu/

- game artificial intelligence
  88

Google Scholar

- "gamification" AND "artificial intelligence"

21.400

- "serious game" "medicine" "medical education" "health" "artificial intelligence"
  404
- allintitle: artificial intelligence game
  575
