# Supplementary material for: The Role of AI in Serious Games and Gamification for Health: Scoping Review
Source: JMIR Serious Games. 2024 Jan 15;12:e48258. doi: 10.2196/48258 (PMC10825760; doi:10.2196/48258)
Supplement: Multimedia Appendix 4 [file games_v12i1e48258_app4.docx]

**Appendix 2: Detailed description of included publications**

| First author, publication year | Disease/ Health topic | Description |
| --- | --- | --- |
| Disease/ Health topic: Motion impairment | | |
| Yeh 2014 | Motion impairment | Development of a non-invasive balance training via an interactive virtual reality (VR) game-based rehabilitation program. 48 subjects with vestibular dysfunction performed VR-adopted exercise programs. Results of pre- and post-training were compared to 36 healthy individuals. To support data analysis, a machine learning tool (Support Vector Machine, SVM) was developed to automatically differentiate between patient characteristics based on quantitative balance indices. Results showed an improvement in balance function with post-training parameters closer to those of healthy controls and good classification accuracy of the SVM between pre-trained patients and healthy controls. |
| Lyu 2019 | Motion impairment | 8 healthy individuals tested a human-robot cooperative control system, in which an EMG-controlled knee exoskeleton is controlled by the human and in an AI-enhanced “assistance-as-needed” approach. Testing was performed with two different AI methods in a visuomotor game environment. Results show improvement in participant score and muscle activation level when using the AI-supported approaches in comparison to EMG-control alone. Using only the AI tech however results in very limited participant involvement and thus low training effect. This indicates the possibility of using the “assistance-as-needed” approach in rehabilitation, e.g. in stroke patients. |
| Nasri 2020 | Motion impairment | Description and training experience of a new dataset for real-time hand gesture recognition. Seven hand gestures are recognized by a deep-learning based method via EMG-signals. These gestures are then processed in a live VR-based 3D game for possible usage in rehabilitation. Testing showed good accuracy in training and in a live test with 15 healthy individuals. |
| Burdea 2021 | Motion impairment | This study presents a new developed game controller for post-stroke motion impairment. When using in a serious game to train upper limb movement, an AI analyses and processes the acquired data and supports in modifying game selection based on baseline and live patient skill level. Games stayed winnable despite different degree of impairment, thus increasing motivation while additionally integrating gamification-like tools like auditive rewards. The system was tested for usability with two healthy individuals, showing good results in ease of use while some technical barriers remain. |
| Zhang 2021 | Motion impairment | Technical description of a textile-based triboelectric sensory system (TENG) for gait analysis (insole) and waist motion capture (belt) to enhance robot-aided lower-limb and waist rehabilitation. The sensory belt is used for control of VR/AR enhanced immersive games for improved engagement in rehabilitation. An artificial neural network is used to process and analyse the acquired data, which then can lead to individualized training programs for rehabilitation. Moreover, integration of the TENG devices to an existent lower-limb rehabilitation robot is possible and promises future usage in clinical practice. |
| Disease/ Health topic: Phantom limb pain/limb absence | | |
| Ortiz-Catalan 2016 | Phantom limb pain/ limb absence | Multicentre single group trial to assess 14 patients with phantom limb pain after upper limb amputation via training phantom motor execution. Based on Machine-Learning aided myoelectric pattern recognition, a virtual limb was added and could be trained via a combination of virtual reality (VR), augmented reality (AR) and serious games were used to create visual feedback. All three scales used as primary endpoints to measure phantom limb pain showed significant decrease in phantom limb pain. |
| Lendaro 2019 | Phantom limb pain/ limb absence | 4 patients with limb absence were monitored over 12 months while training phantom motor execution. Based on Machine-Learning aided myoelectric pattern recognition, the in-home use was tested with four different training support tools – both a VR and AR environment and two Games. The study focused on ethnographic analysis of user behaviour. Results indicate good functioning in principle, adherence and related outcomes however depends on many patient-individual factors such as perceived need, social context, and technical requirements, cumulating in the importance of analysing “user types”. |
| Kristofferson 2021 | Phantom limb pain/ limb absence | Eight patients with upper limb absence were separated into two groups to improve control of their prothesis: One trained by a conventional approach, the other by a serious game-based approach. Training data were integrated in a machine-learning controlled prothesis with the aim to improve EMG output quality and degrees of function in comparison to the patients’ own prothesis. Results in this limited sample size (only 4 patients finished the study) show no overall significant improvement in the game-based training and better results in using the own prothesis. |
| Disease/ Health topic: Cognitive impairment | | |
| Valladares-Rodriguez 2018 | Cognitive impairment | Pilot study to demonstrate the possibility of early detection of mild cognitive impairment. A set of serious games (Panoramix) is used to assess different cognitive features cumulating in an overall analysis of cognitive function based on existing clinical standards. Machine learning was then used to test automatic discrimination between healthy individuals and patients with MCI or Alzheimer Disease (AD). Therefore, a first cohort study with 16 individuals, suffering from MCI or AD was conducted. Results show good construct, criterion and external validity of the games set in comparison to existing diagnostic standards. Furthermore, the pilot testing showed a positive participants perception, while gaining further insights into possible usability improvement. Prediction accuracy improved by machine learning methods in comparison to logistic regression. |
| Valladares-Rodriguez 2019 | Cognitive impairment | Based on the preliminary findings of the study mentioned above, a further usability study of Panoramix within patients and health professionals was conducted. Player experience of 74 senior users, either suffering from AD, MCI or healthy controls, and 15 test administrators was analysed. Results show high accessibility and usability in both groups. Additionally, patients showed high willingness to participate in using these tools. Further diagnostic validation is planned to follow in further studies. |
| Jung 2019 | Cognitive impairment | Proof of concept of 12 post-stroke patients with mild cognitive impairment, who use a set of six mobile games (Neuro-World) to self-administer the assessment of cognitive impairment level after stroke and at 3-months follow-up. Aim of the study is to validate, that the game performance can reflect scores on the mini mental state examination (MMSE) as parameter for cognitive function assessment widely used in clinical practice. Machine learning models are used to estimate the corresponding MMSE score based on game results. Results show only a 5.75% normalized root mean square difference to a professionally conducted MMSE with good responsiveness for changes in the study period. Furthermore, testing showed positive usability and willingness of participants. |
| Disease/ Health topic: Rheumatoid arthritis | | |
| Varga 2021 | Rheumatoid arthritis | Development of a serious game for hand rehabilitation in patients with rheumatoid arthritis. Neural networks are used for processing the app data, with machine learning testing the accuracy of hand movements and thus adapting game difficulty as well as individualized feedback at the end of the game. A first pilot testing with 7 healthy individuals showed high accuracy of the machine learning algorithm, whereas clinical benefits could not be measured. |
| Varga 2022 | Rheumatoid arthritis | In a follow-up study using the above-mentioned serious game for hand rehabilitation, 10 patients with rheumatoid arthritis confirmed curiosity and willingness to use the application, while physical and psychical effort was rated rather easy. Disease-related functional improvements were not measured. |
| Disease/ Health topic: Cancer | | |
| Good 2014 | Cancer | Open online game to collect knowledge over the importance of specific genes to predict breast cancer survival. Over 1000 players, with and without expertise (experts = self-reported to have either a PhD or MD and knowledge of cancer), selected a set of five genes predictive for long-term survival based on their knowledge, in-game text descriptions and/or random clicking against a computer player. Gene selections were processed by machine learning to identify the best prediction models. Results showed this crowdsourcing approach being effective in collecting knowledge in the expert’s subgroup, resulting in no significantly different performance than clinically established gene sets. |
| Disease/ Health topic: Attention-deficit/hyperactivity disorder | | |
| Keshav 2019 | Attention deficit hyperactivity disorder | 7 students with attention deficit hyperactivity disorder (ADHD) used a smartglass to assess ADHD severity through playing an Attention-Related Augmented-Reality game, designed as social-emotional communication aid. Artificial intelligence is used to analyse video, audio, affective, and behavioural data and provides users with in-game rewards based on their performance. The study showed significant correlation of the game score to validated clinical assessments for ADHD. |
| Disease/ Health topic: Other | | |
| Pinto 2019 | Other | Presentation and pilot testing of GameAAL, an at-home architecture consisting of ambient sensors, biomedical monitoring, and neurocognitive games to provides cognitive stimulation and remote monitoring of health and activity parameters. Data is then analysed by machine learning and feedback to the user including gamification elements like badges or benefits for real-life events is provided. Goal is to facilitate prevention of cognitive and physical decline and early detection of behavioural changes and pathologies. A first pilot-testing with 11 elderly subjects showed good usability of the system. |
